# Rest as a Therapeutic Agent*Read at Brazos Valley Medical Society, May, 1906.

**Published:** 1906-10

**Authors:** L. M. Barnes

**Affiliations:** Thorndale, Texas


For Texas Medical Journal.

### Rest as a Therapeutic Agent.*

BY L. M. BARNES, M. D., THORNDALE, TEXAS.

This is a very complex and complicated physiological state, and includes mental, physical and the moral being. When we remember that every movement is an expenditure of force, or energy, and that life is molecular motion and death is just the opposite, how strange that rest so similar to death is classed as one of the therapeutic agents. It is classed with drugs as a means applied to the cure of disease.

The social nature of man and his consciousness make it complex with his environment, and mental relations to the same make it difficult, and the least successful plan of treatment the average doctor has to direct. Mother, father and a host of friends prevent the treatment from being successfully carried out. The phenomena of our being is absolutely wonderful.

Man is wonderfully and fearfully made, if you please. I quote from sacred writ. Beginning with infancy, and the study of its habits, is an evidence of the need of rest. In a normal, healthy state they sleep twenty hours out of twenty-four; and, in fact, they do very little except eat, digest, and sleep, and sleep is properly a state of rest. According to Billings' Dictionary: "Sleep is that state in which the functions of sensation and volition are suspended while the vital functions retain their activity in a modified degree."

Dr. S. Weir Mitchell, of Philadelphia, has suggested a treatment for a certain class of patients whom he designates as not having any well-defined, organic disease, who may successfully be treated without drugs, and Dr. Hobart Amory Hare, of the same city, includes this treatment in his excellent hand-book of therapeutics, calling it the rest-cure. I do not wish to suggest any criticism of the treatment, but rejoice to find it a text for this subject, and may I not suggest that not only a page or two, but a volume of considerable size can be written on the subject that would be of untold benefit to humanity and to the medical profession in particular. Drugs, patent medicines, secret nostrums and pharmaceuticals have books, pamphlets, journals, and both the secular and religious press, with posters, almanacs, calendars, etc., which

*Read at Brazos Valley Medical Society, May, 1906.

suggest certain drugs to all who are sick or think they have a disease. The rest cure is not limited to any class of patients, but it should be enjoined upon all patients, and carefully enforced upon them, whether it be an infant at the mother's breast or a child tottering about the room, a youth in school or spending late hours at night, full manhood with the carking cares of life or the octogenarian retrospecting the past life.

Rest is said to be nature's restorer, and of a truth we see its healthful effects and feel its wonderful truths when all our being has been active for a period, and a rest is secured. Men are said to sleep in the din of battle and the quaking of the earth will lose its force when the physical has gone to its limit of endurance.

If those who are said to be in perfect health and capable of physical and mental and moral activity require periodical rest, is it not logical that the diseased need it a hundredfold more?

Rest is a state in which the reconstructive powers are manifested more clearly than any drug. I have seen the loss of weight and general inanition from a restless patient with a felon, and a quiet, restful sleep restore mental, physical and moral energy absolutely to a wonderful extent in ten hours. Would you expect a case of typhoid fever, pneumonia, dysentery, a fractured limb, or a gaping wound to make happy recovery without rest? There is something in nature, and I speak reverently of her hidden powers, which is medicine to the sick. If you please, homeopathy, Christian science and many other theories depend upon rest for the cure of the afflicted, and we are the losers of many patients who could properly be treated if we would study more closely the laws governing active life forces, and rest as a remedial agent.

Did you ever figure out how many thousand heart beats are saved in twenty-four hours in a state of rest, or counted the gain of strength by slowing the respiration in a state of rest? Can it be computed? Do we figure the time it takes to digest foods, and the rest the baby's stomach gets with an anxious mother as its nurse; do we so prescribe our drugs that a proper limit of time be granted the patient as much rest as they would take if they were in health? These are questions of moment to you and me, and I trust will prove a benefit to the sick. What takes place in the brain, and nervous system, during sleep and why do the lungs and the heart require a rest, and what the secretions store up for use in our active state is not fully understood, but, doubtless, the anti-toxines which render immunity to disease possible is a plausible theory. Excretion, secretion, respiration, the circulation and the entire being is regenerated most perfectly during a state of repose.

In conclusion, I will say that in order to appreciate the subject you must visit some of our modern sanatoria where quiet and comfort appear the highest aim, and the surroundings with the trained nurses are so inviting to those who are afflicted. It makes one feel like he would rejoice to see every cottage home a sanatorium and every mother a trained nurse, instead of the ordinary experience of poor hygiene and a house filled with noisy, sentimental friends. I give you one example in which rest was my last resort. I had a lady patient some eighteen years ago who had typhoid fever, and on the thirty-second day of the fever she had hemorrhage of the nose for ten or twelve hours, in spite of all I could do. Finally I sent for a doctor. Before he arrived, she was so exhausted that she fell asleep, and when he came he suggested to let her rest, and possibly nature would do the work. I submitted, and for four hours we quietly waited until she awoke. The hemorrhage in the meantime ceased and her fever subsided, and she made an uneventful recovery. The doctor taught me something.

